# Differences in survival and phenotypic traits of curly birch preserved by heterovegetative propagation: a case study from Central-East Europe

**DOI:** 10.1038/s41598-021-87508-0

**Published:** 2021-04-13

**Authors:** Ivana Sarvašová, Róbert Sedmák, Denisa Sedmáková, Ivan Lukáčik

**Affiliations:** 1 Arboretum Borova hora, Technical University in Zvolen, T. G. Masaryka 24, Zvolen, 960 01 Slovak Republic; 2 Faculty of Forestry, Technical University in Zvolen, T. G. Masaryka 24, Zvolen, 960 01 Slovak Republic

**Keywords:** Forest ecology, Forestry

## Abstract

Curly birch (*Betula pendula* Roth. var. *carelica* [Merklin] Hämet-Ahti) is a disappearing representative of the *Betula* genus facing a regeneration failure in a large part of its natural distribution in Europe. The unique long-term study of clonal replications originating in heterogeneous environments enabled the evaluation of long-term survival and phenotypic stability of progenies in seed orchard to assess the conservation and commercial potential of heterovegetative propagation. Seventy-eight geographic sources (95 clone origins) representing the south distribution edge in East-Central Europe were analysed for species variation in survival, growth form, bark colour, and stem quality of parent trees and their vegetative progeny, and the effects of four parental site origin characteristics. The survival rate was 73% after 28–33 years of growth. Retention of curly-grained wood was high, the curly-grained wood structure is heritable and thus clonally efficiently achievable (only 3.5% of grafted individuals showed no occurrence of figured wood structure). The phenotypic expression of curliness manifested on the trunks as bulges, stem growth forms (tree/shrub) and stem technical quality showed a lower degree of stability (coincidence) between the parent trees and heterovegatively propagated progenies. Despite this, the conservation potential of seed orchard is very high, especially when stabilization of the stem growth forms affecting the survival and commercial value of progenies can be probably achieved by a more careful selection of scions. Overall, heterovegetative orchards seem to be a very promising method for the long-term conservation of curly birch populations, which, in addition to their great biological and ecological value, have considerable commercial potential.

## Introduction

Curly birch (*Betula pendula* Roth. var. *carelica* [Merklin] Hämet-Ahti)—a rarely occurring tree—has the natural range limited to the middle part of silver birch (*Betula pendula* Roth.) distribution^1^. Its natural range is discontinuous with islets and mosaic-like occurrence, usually in separated micro populations (2–3 ha), at the northeastern distribution border even of 5–10 ha large^2^. It is mainly distributed in northern Europe with the northernmost presence in the vicinity of Lake Onega and the Karelia Plateau (Finland, Russia) and the easternmost at Jaroslavl. In the southwest direction, it is spread in the Baltic countries, Belarus, Poland, southern Sweden and Norway, with the most western occurrence recorded at Ingolstadt in Germany and Denmark^3^. In Slovakia, the southern distribution edge is present (the most southern occurrence in Slovakia is found in the Zemplin's mountain range^4^). Sporadically, it also occurs in the Low Beskids in the Czech Republic^5^. Its patterned wood structure makes it a highly valuable wood, which is used mainly for producing various gifts, furniture, musical instruments as well as for decorative veneer or small items.

At the same time, the curly birch is a disappearing representative of the *Betula* genus and belongs to endangered plants registered in the Red Book of Plants of the Republic of Karelia and to protected species of the whole Russian Federation^6^. It is facing a regeneration failure in a large part of its natural distribution, likely due to an interplay of its high light requirements and low degree of seed germination. Moreover, the discontinuous distribution range impeding gene flow among isolated subpopulations and the changes in land use causing the reduction of suitable habitats are important factors as well. The ongoing climate change manifested in Slovakia by higher temperatures and prolonged periods of spring droughts make the natural regeneration of curly birch even more difficult. No viable regeneration within the entire geographical range of curly birch was pointed out by Vetchinnikova and Titov^7^. Thus, urgent protection actions are highly advisable.

**Figure 1:**
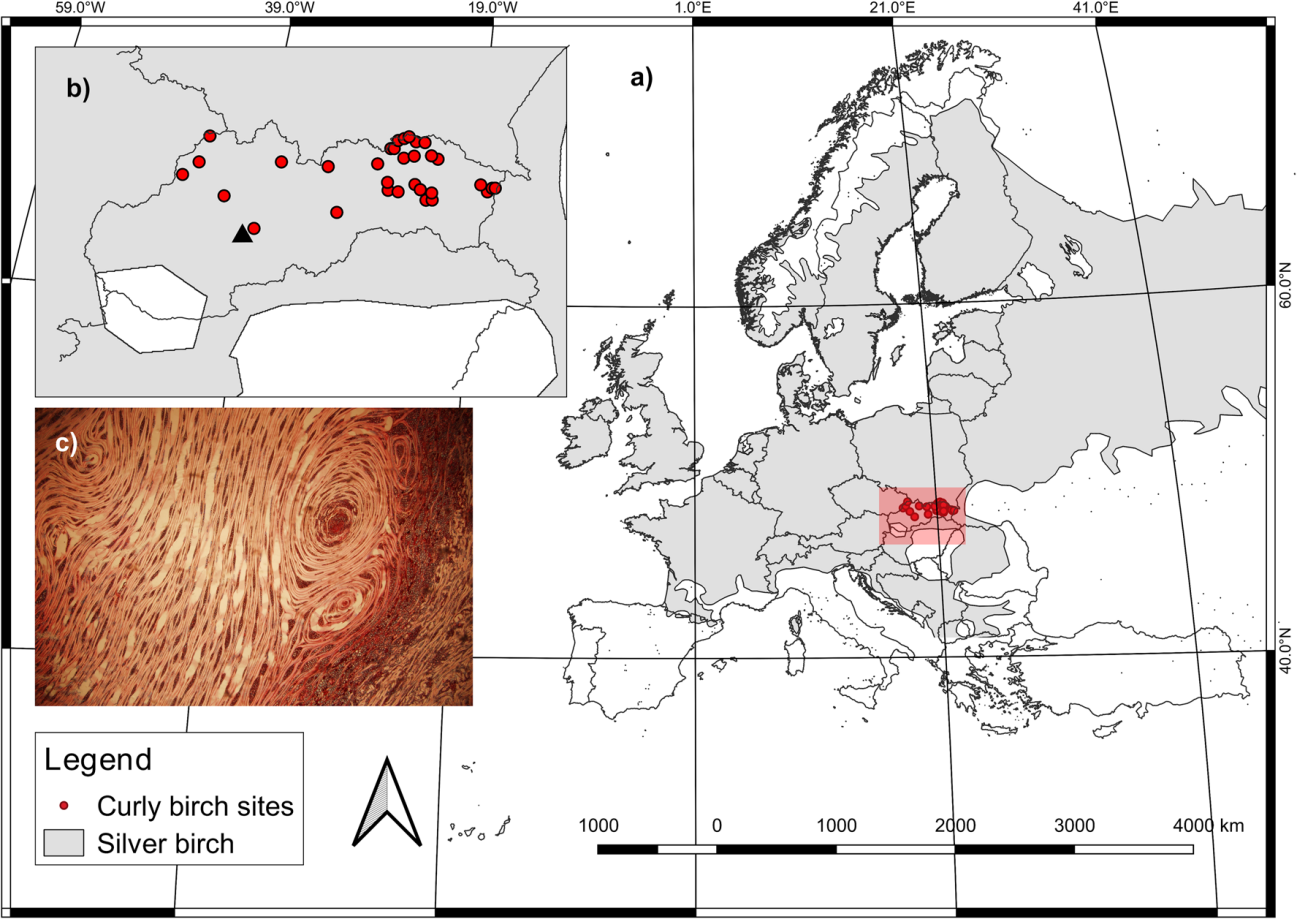
(**a**) Distribution of silver birch^25^ and (**b**) location of initially established seed orchard (triangle) and curly birch sampling sites (circles) corresponding with its natural distribution, showing the most frequent occurrence of curly birch in the north-east part of Slovakia and its absence in the south and central areas (**c**) tangential micro-section of the specimen showing the specific curly grained wood pattern (photo by D. S.).

Curly birch is easily clonally propagated. To understand the formation of figured wood, already in the twentieth century, several authors have developed a method of vegetative propagation to establish seed orchards to produce hybrids with the highest possible proportion of bulges on single stems. These orchards represent also very valuable gene archives for conservation purposes (Fig. 1). Under special conditions for obtaining pure clones, it is possible to propagate curly birch by summer cuttings, and even by winter cuttings, but with lower success^8^. An established simple and prospective propagation method of tissue culture^9–13^ has been shown to have a hormonal imbalance and structural disturbance of the tissues due to exclusion of epigenetic impacts—environmental factors^14^ and possible occurrence of undesirable somatoclonal changes^15^.

**Figure 2:**
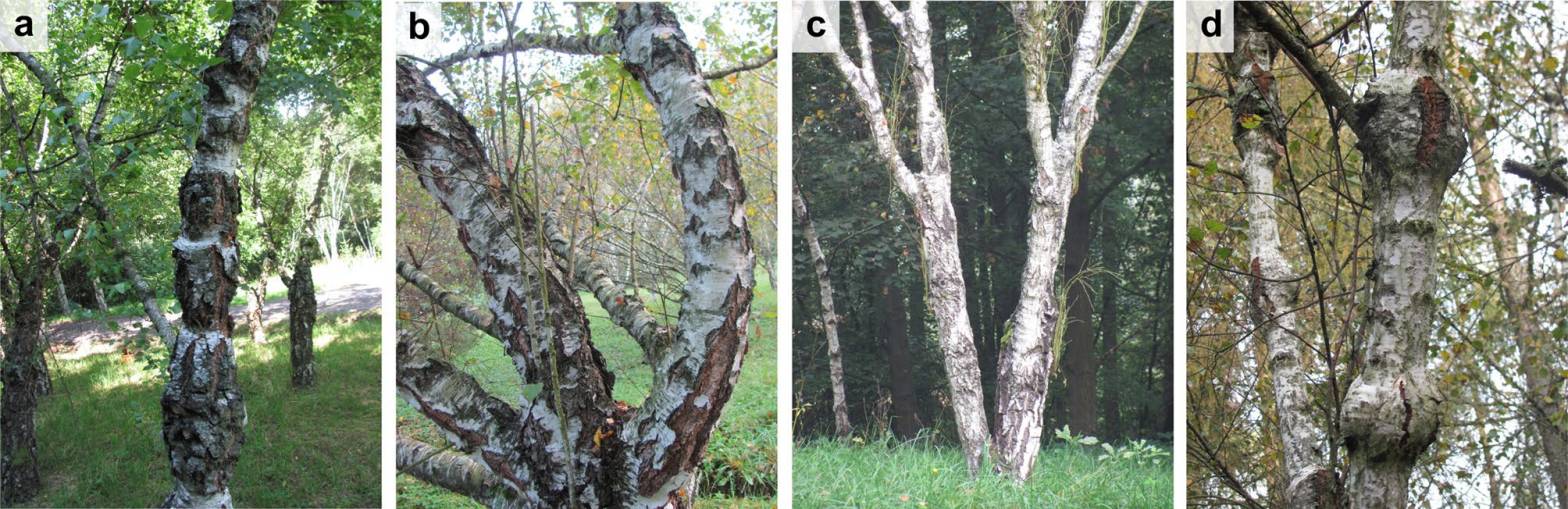
Appearance of curly birch in seed orchard ʻŠírinyʼ. The shape of bulges corresponding to four defined types: (**a**) ring, (**b**) dense, (**c**) shallow, (**d**) sphere (photo by I. S.).

The most suitable propagation forms for the valuable origins of curly birch include clonal propagation by grafting, also called heterovegetative (or xenovegetative) propagation, under the conditions already established by Lyubavskaya^16^. It is a type of side graftage, where the size and thickness of the scion, the time of grafting and the secured dormancy of the grafts play an important role. The individuals with tree form and external presence of shallow and dense types of bulges are commercially very valuable as they can be utilized for the veneer or precious furniture production. The individuals with tree form and other types of bulges and shrub form with any type of bulges are considered less valuable, suitable to produce various small decorative items (although they still have a very good commercial potential, Fig. 2).

Despite the high commercial wood value and conservation needs, the stem quality, form of bulges or their external manifestation after the transfer from parent trees to clone archives have not yet been studied in detail. In seed orchards, after five years of planting, up to 40–60% of individuals in young plantations showed bulges^8,17^. Faster growing individuals (i.e., taller individuals) had a lower number of bulges than slower-growing ones. Václav^8,17^ and Kärkkäinen et al.^18^ consistently point out that grafted tree individuals in seed orchards are usually smaller and grow more slowly than at the same time established seeded plantations of curly birch hybrid progenies.

The type of figure or other ornamental patterns in the wood of clonally propagated progenies in seed orchards is genetically controlled, as confirmed for curly poplar and curly black walnut, but the inheritance of figured grain wood pattern may not be simple^19,20^. Studying the inheritance, the ways of transmission of qualitative features in clonal propagation, and their description might be the first step toward understanding the creation of commercially valuable figured wood. Recently, Vetchinnikova et al.^15^ state that no single case is found that the wood structure of the rootstock would penetrate the grained wood structure of the scion when grafting the curly birch. Each part of the new individual creates its wood structure, while at the same time the intensity of growth corresponds to the relationship between the rootstock and the scion. The growth intensity of both parts combined into one unity should be equal, especially for the sake of successful cultivation and conservation of curly birch genetic resources. Uneven growth (slower growth of the scion and faster growth of the rootstock, and vice versa) is often the reason for a later death of the individuals in the seed orchard or the gene pool (reserve) of such a rare taxon.

Novitskaya et al.^21^ and Vetchinnikova et al.^13^ point to interesting facts about the presence of a latent period in the formation of a figured grain wood. Authors describe the partial production of grained wood (only on a ½ or ¼ of the stem circumference), whereas the formation of figured wood is always located on a side of a well-lit part of the crown of an individual. In grafting, respectively in the transfer of bark of *B. pendula* var. *carelica* on the rootstock of *B. pendula*., the effect of figured wood formation is strengthened as stated by the authors. Formation of figured wood is both genetically and environmentally controlled^14^. Growth, survival or formation of a wooden figure of *B. pendula* var. *carelica* could sensitively respond to transfer from the naturally most southern occurrence of local origin. For instance, the survival rate of the *B. pendula* progeny is the highest at the local origin of parent tree site, whereas too distant transfer from local origin decreases the survival^22^.

In Slovakia, the research effort concerning the curly birch has been mainly aimed at saving the scarce and valuable material by vegetative propagation. It began in the late 1970s with the national systematic survey of locations for the presence/absence of *B. pendula* var. *carelica*. Subsequently, seed orchard established in randomized block design represents one of the best-preserved gene pool of curly birch in Slovakia and serves as a base for long-term research.

Our main aim in this study is to evaluate the survival and stability of phenotypic manifestations of vegetatively produced progenies in the established seed orchard, particularly:

i. To evaluate the survival of the new, heterovegetatively established, population of the *B. pendula* var. *carelica* in general and according to growth forms and other important traits of parent clones (shape of bulges, stem technical quality, bark colour) to reveal potential associations among them.
ii. To tests and find out whether the individual stature characteristics (tree or shrub growth form), the shape of bulges, stem technical quality and bark colour of parent clones correspond well with phenotypic manifestations of the vegetatively produced progenies, evidencing good stability and sufficient preservation in the seed orchard.
iii. To explore the statistical relationships among survival rates and proportions of shrub/tree growth forms of progenies and selected environmental characteristics of parent sampling sites (altitude, distance and direction of transfer from parent site to the orchard) to confirm or reject the hypotheses about the potential environmental conditionality of progeny survival or growth form.

The general aim of the study is to evaluate the level of long-term survival and phenotypic stability of progenies important both in terms of curly birch conservation efforts or in terms of establishing its commercial plantations and at least partly to fill an information gap in this regard utilizing the rare long-term dataset.

## Results

### Curly birch population and progenies

The majority of progenies originated from parent clones of tree growth form and 92% had white bark colour. Concerning the stem technical quality, 27% of parent clones had ring, 22% dense, 39% shallow, and 12% sphere shape of bulges. Propagated material originated from 16 orographic units (78 localities) in Slovakia and was transferred mainly from the north-east to the south-west direction (Table 1). The average transfer distance was 133 km and the mean altitude of clone origin was 514 m asl. Clonal propagation by grafting, also called heterovegetative propagation, has not affected curly grain wood formation in birch. Only 3.5% of grafted individuals showed no occurrence of patterned (grained) wood structure apparent on the surface of stem or branches. The average survival rate of grafts was 73%. Since the establishment of clonal seed orchard during 1982–1988, 658 out of originally grafted 896 mainly tree individuals (95 clones) were present (alive) according to the census conducted in 2015.

**Table 1:** Geographic units with natural occurrence of curly birch in Slovakia including the numbers of clones (origins) and the total number of vegetatively propagated individuals used in the study (for details on orographic units see a geomorphological division of Slovakia—https://en.wikipedia.org/wiki/Geomorphological_division_of_Slovakia).

	Orographic unit	Area	Sub-province and province in the Carpathians	No. of clone origins	No. of progenies	Altitude (m asl)	Mean transfer distance (km)	Transfer direction	Bedrock^23^
1	Beskidian Piedmont	Low Beskids	Outer Eastern	13	98	320–420	245.0	SW	claystone, sandstone, flysch
2	Busov	Low Beskids	Outer Eastern	4	42	500–520	145.0	SW	greywacke/arkosic sandstone, sandstone, claystone, variegates shales, flysch
3	Laborec Highlands	Low Beskids	Outer Eastern	1	11	350	240.0	SW	calcareous sandstones, siltstones and claystone, rare slumps and limestones, claystone, sandstones, flysch
4	Ondava Highlands	Low Beskids	Outer Eastern	36	325	420–550	188.3	SW	claystone, sandstone, flysh
5	Žiar	Fatra-Tatra	Inner Western	1	9	575	48.0	ESS	limestones, dolomites, locally shales and sandstones
6	Torysa Highlands	Lučenec-Košice Depression	Inner Western	1	10	600	170.0	SWW	variegated conglomerates, sandstones and shales, local evaporites and volcanic rocks
7	Slanské Hills	Matra-Slanec	Inner Western	13	135	350–675	180.4	SWW	propylized andesites and andesite porphyry of central zones
8	Black Mountain	Slovak Ore Mts	Inner Western	3	29	560–580	143.1	SWW	limestones, dolomites, locally shales and sandstones calcareous shales, shales, sandstones and quartzites
9	Revúca Highlands	Slovak Ore Mts	Inner Western	2	74	575	85.0	W	granites, grandiorites, tonalites
10	Volovec Mountains	Slovak Ore Mts	Inner Western	2	15	350	102.0	W	graphite and sericite phyllites, metasandstone, metamorphosed silicic volcanic
11	Zvolen Basin	Slovak Ore Mts	Inner Western	2	5	350	0.0	no	pyroxenic and ambifolic-pyroxenic andesites
12	Skorušinské Hills	Podhale-Magura	Outer Western	3	31	900	93.0	S	shales, marls, sandstones, conglomerates, mostly flysch
13	Šariš Highlands	Podhale-Magura	Outer Western	5	44	600	152.0	SW	variegated conglomerates, sandstones and shales, local evaporites and volcanic rocks
14	White Carpathians	Slovak-Moravian Carpathians	Outer Western	1	11	500	86.0	SE	propylized andesites and andesite porphyry of central zones, shales, marls, sandstones, conglomerates, mostly flysch
15	Javorníky	Slovak-Moravian Carpathians	Outer Western	2	8	550–600	82.0	SE	claystone, sandstones, flysch
16	Čergov	Eastern Beskids	Outer Western	6	49	480–650	165.8	SW	greywacke/arkosic sandstone, sandstone, claystone, variegates shales, flysch

### Effect of the parent clone

#### Survival of progenies

**Figure 3:**
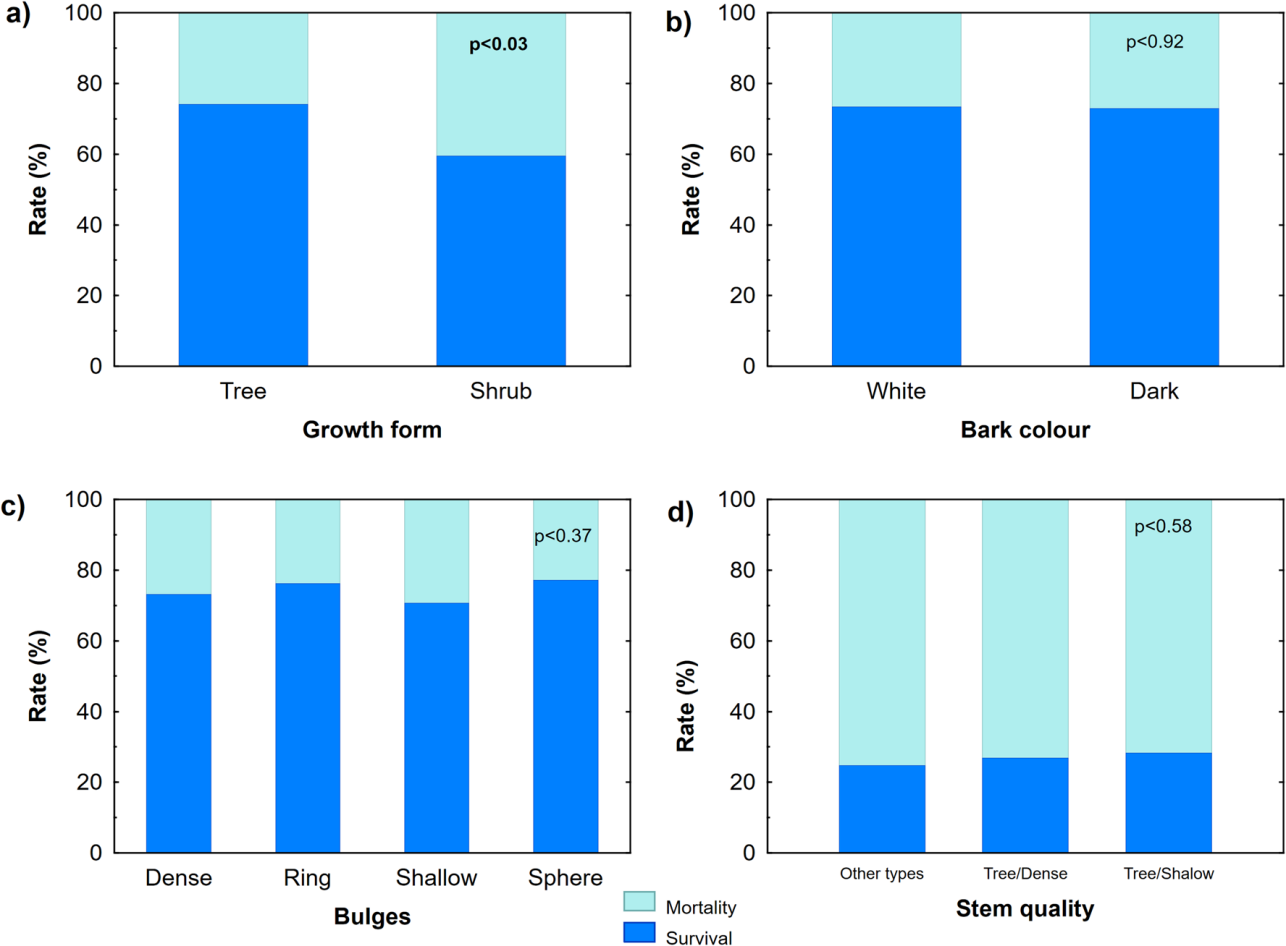
Survival and mortality rate of the curly birch progenies according to characteristics of parent trees displayed on x-axis: (**a**) growth form, (**b**) bark colour, (**c**) type of the bulges and (**d**) stem quality; the percentage of survival rate of progenies—dark blue, percentage of mortality rate—light blue.

Progenies from parent clones of shrub growth form had a significantly higher degree of mortality (lower survival rate) than progenies coming from clones of the tree-like form (confirmed by *Chi*-square test *p* < 0.03; Fig. 3a). Particularly, the combination of shrubs with a shallow form of bulges had a lower survival rate (43%) compared to others, although differences in survival/mortality rates according to bulge forms were not significant (*p* < 0.10). We also detected no relationship between survival rate of progenies and bark colour (p < 0.92; Fig. 3b) or type of bulges (*p* < 0.37; Fig. 3c). Progenies from high-quality parent trees with the most valuable form of bulges (dense or shallow), suitable for veneer processing, did not differ in the survival rate between each other and to all other remaining types (*p* < 0.58; Fig. 3d).

#### Correspondence of progenies with parent clones

**Figure 4:**
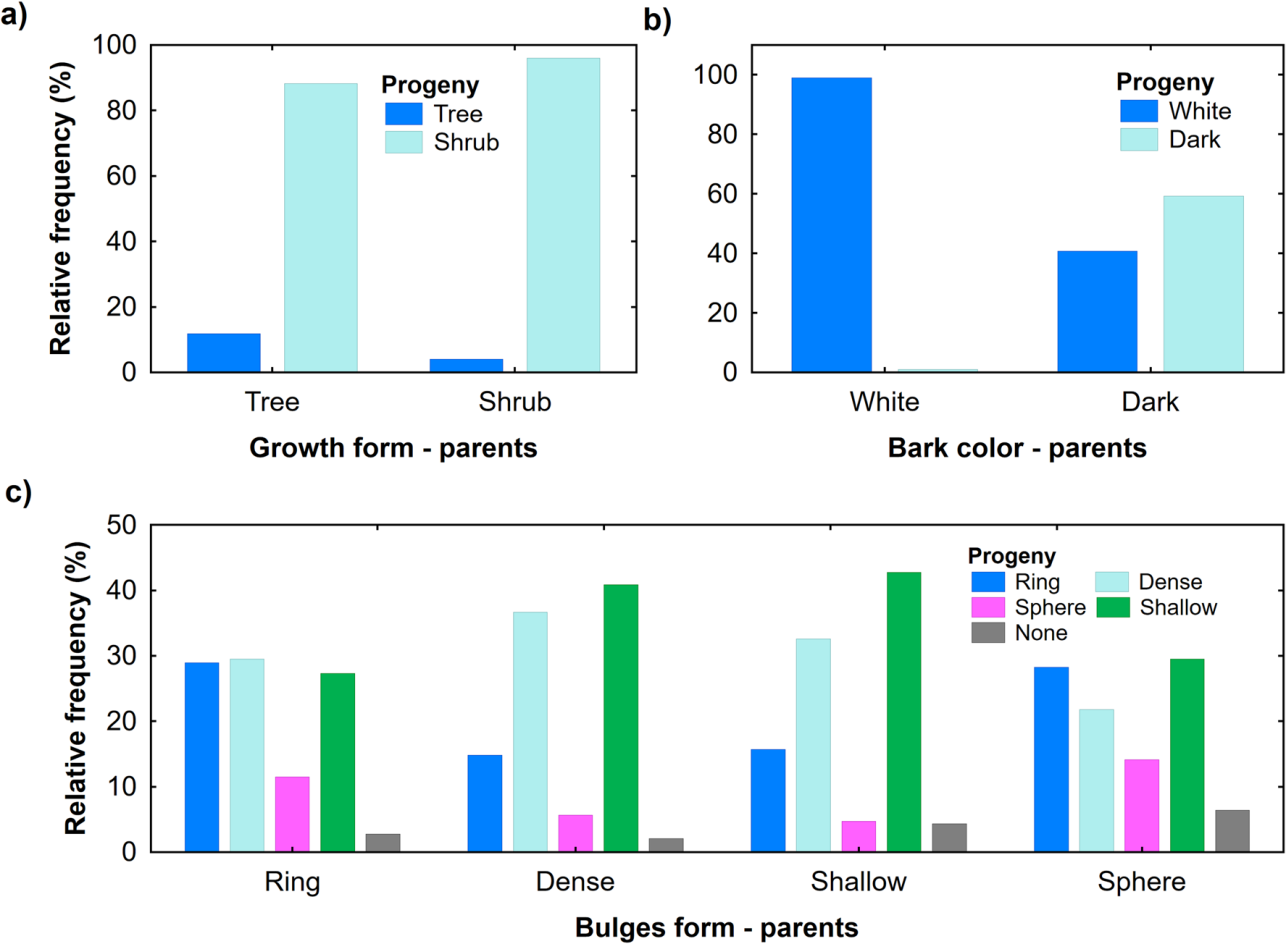
Correspondence of the curly birch progenies according to characteristics of parent clones displayed on x-axis: (**a**) growth form, (**b**) bark colour, and (**c**) shape of the bulges, values on y-axis show relative frequencies of progenies in selected parent categories.

Confirmed by the overall *Chi*-square statistic among the selected features, the highest coincidence between progenies and parent clones was in the bark colour and the highest discrepancy was in the growth form and item combinations (Tables 2, 3). Ninety-nine per cent of progenies corresponded to the white bark colour of parent clones and nearly sixty per cent with the black bark colour of parent clones (Fig. 4b). In contrast, only about twelve per cent of progenies originating from clones of tree growth form remained trees (Fig. 4a). Concerning the shrub growth form, the majority (96%) of progenies corresponded with their parent clones, though four per cent of grafted individuals originating from shrubs displayed tree growth form. The progenies with the dense and shallow shape of bulges showed similar patterns of relative frequencies across types of bulges of parent clones. The progenies with the dense and shallow shape of bulges revealed the most stable form as they showed the highest frequencies in the respective (corresponding) types of bulges with parent clones (Fig. 4c).

**Table 2:** Correspondence of the morphological traits and technical quality between parent clone and progeny plants (Bark colour—dark or white; Small items—shrubs with any type of bulges and trees with ring or sphere shape of bulges; Bulges shape—ring, dense, shallow, or sphere; Veneer—tree growth form with the external presence of shallow and dense types of bulges; Growth form—tree or shrub).

Variable	Chi-squares	Degrees of freedom	*p*-level
Bark colour	309.25	1	0.000
Small items	69.74	42	0.005
Bulges shape	40.51	12	0.000
Veneer	6.36	4	0.174
Growth form	1.45	1	0.229

**Table 3:** Coincidence and discrepancy rates of the morphological traits and technical quality between parent clone and progeny plants. (t-value—student T-test of deviances of discrepancy rates from zero; Bark colour—dark or white; Small items—shrubs with any type of bulges and trees with ring or sphere shape of bulges; Bulges shape—ring, dense, shallow, or sphere; Veneer—tree growth form with the external presence of shallow and dense types of bulges; Growth form—tree or shrub).

Variable	Coincidence	Discrepancy	t-value	*p*-level
Bark colour	95.7	4.3	5.41	0.000
Small items	6.5	93.5	95.76	0.000
Bulges shape	35.5	64.5	33.95	0.000
Veneer	41.2	58.8	30.65	0.000
Growth form	15.0	85.0	60.95	0.000

The possibilities in curly birch utilisation to grow high-quality trees for the veneer and precious furniture production by clonal propagation seem to be low. There is a certain degree of correspondence in the clonal propagation of trees with high-quality stems/logs (trees with dense and shallow bulges suitable for veneer production), but this correspondence is not statistically significant (Table 2). The combination in low correspondence of tree growth form, but stable production of dense and shallow bulges resulted in relatively low differences between coincidence and discrepancy values (Table 3). On the other hand, less valuable branch wood of trees and wood of shrubs can be utilized to produce various small decorative items. The correspondence of progenies with parent clones in the suitability for production of various small wooden items (wood of smaller dimensions or with knots) seem to be relatively high (Table 2), pointing to stability in shrub growth form correspondence of progenies. The unstable correspondence of the ring and sphere shape of bulges probably resulted in the highest discrepancy value for small items (Table 3).

### Effect of parental environment

#### Survival of progenies and their growth form

**Figure 5:**
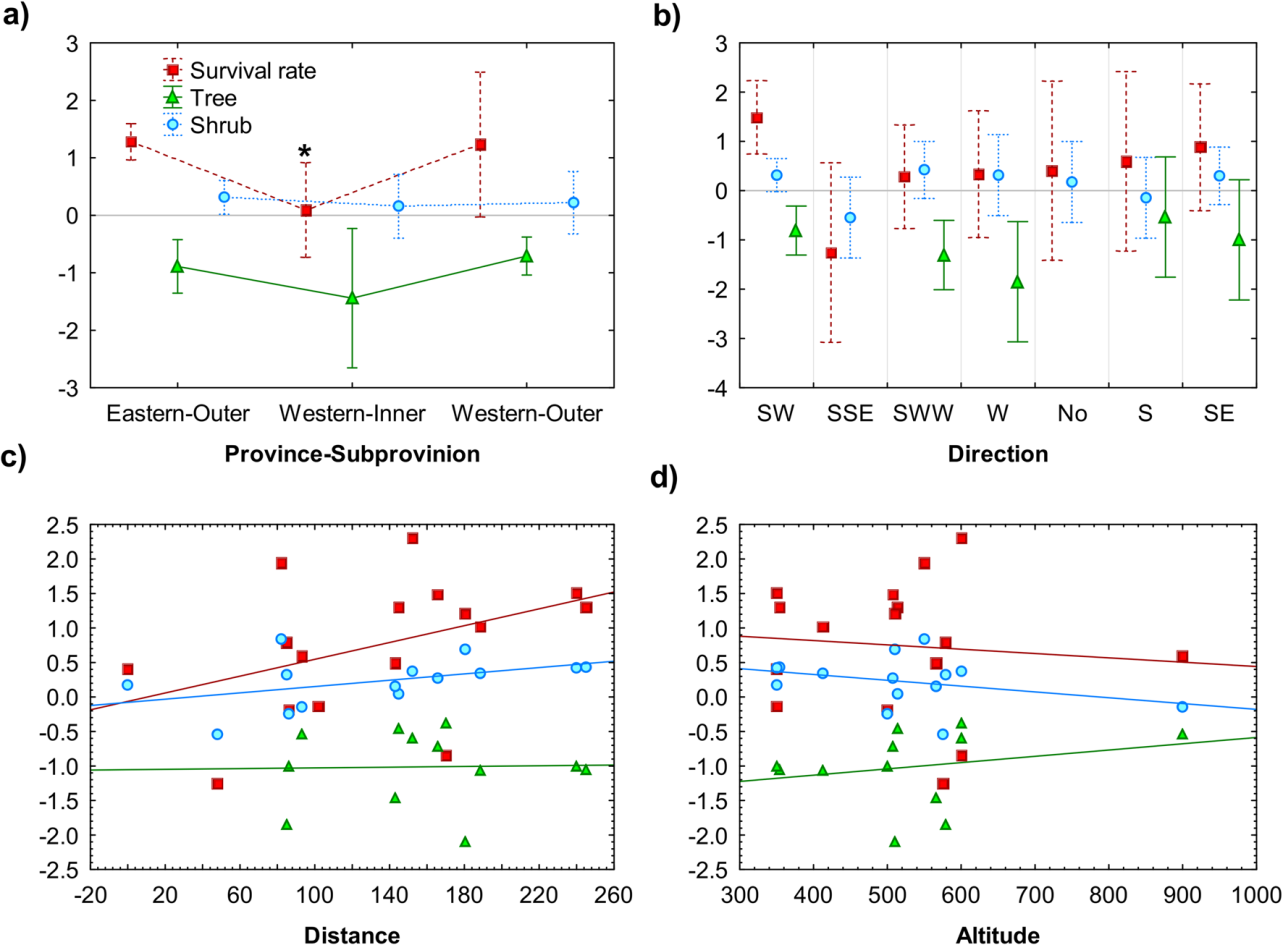
Survival and growth form of the curly birch progenies according to characteristics of the parental environment: (**a**) geomorphological unit, (**b**) transfer direction, (**c**) transfer distance (km), and (**d**) altitude (m asl). The survival rate, percentage of trees and shrubs are expressed on the y-axis as logit transformed values, vertical bars denote 0.95 confidence interval, *- marks significantly different means.

The parental environment had a weak effect on survival rate and proportions of growth form of curly birch progenies at the time of field survey (app. 30 years since planting). Only, one environmental variable—sub-province—had a significant effect on the survival rate of curly birch progenies. Based on ANOVA results, progenies of parent trees originating from geomorphological sub-province Inner Western Carpathians had a significantly lower survival rate (F_(2,13)_ = 3.82, p < 0.05; Fig. 5a) than progenies from the other two sub-provinces. Also, the proportion of progenies displaying tree growth form was lower from sub-province Inner Western Carpathians.

The direction of parent tree locality toward the clonal seed orchard seems to be unimportant (Fig. 5b). Although, grafts from parent trees transformed from northwest to south-east (the SSE direction) corresponding to the Fatran-Tatra region of the Inner Western Carpathians had the lowest survival rate. The survival rate of the grafts from parent trees transferred from north-east to south-west (the SW direction) was the highest. Though, the effect of transfer direction was not-significant (F_(6,9)_ = 2.17, *p* < 0.14). Neither, was the effect of transfer direction significant for growth form of progenies (F_(6,7)_ = 1.21, *p* < 0.40—shrubs; F_(4,7)_ = 1.34, *p* < 0.34—trees).

The survival rate of progenies and the proportion of progenies with shrub growth form increased with the distance of parent tree locality from seed orchard location, showing weak insignificant correlations (r = 0.42, *p* < 0.11—survival rate; r = 0.44, *p* < 0.11—shrubs; Fig. 5c). Distance did not influence the proportion of progenies with tree growth form. The altitude of parent trees locality had a weak negative insignificant relationship with proportion of shrub growth form of progenies (r =  − 0.33, *p* < 0.25) and weak positive insignificant relationship with proportion of tree growth form (r = 0.24, p < 0.45). The altitude of parent trees locality showed no relationship with the survival of curly birch progenies (Fig. 5d).

#### Progenies with high stem quality

Parent curly birch trees from which timber can be utilized for veneer or precious furniture production (tree growth form with the external presence of dense and shallow shape of bulges) originated from eight out of sixteen orographic units, namely Busov, Laborec Highlands, Ondava Highlands, Slanské Hills, Black Mountain, Revúca Highlands, Javorníky, and Čergov (Table 1). Within these units, the proportion of high stem quality parent trees selected for vegetative propagation ranged from 34 to 100% (63% on average). The highest proportions originated from the Low Beskids region, the furthest region in the north-east direction from the seed orchard. The relative proportion of high stem quality progenies decreased in all above mentioned orographic units, significantly in six units (Busov, Laborec Highlands, Ondava Highlands, Slanské Hills, Revúca Highlands, Čergov). Decreases ranged from − 10 up to − 88% (− 41% on average). Consequently, the average occurrence of high-quality progenies was 22.5%. Thus, the occurrence of high quality stems among progenies was 3 times smaller than in the parent tree set and only one-third of progenies retained the high-quality stem on average.

On the other hand, in seven out of eight orographic units without the presence of high stem quality parent clone origins, we recorded high stem quality progenies. The proportion of high-quality progenies from their total number ranged from six to 20% (13% on average) and was significant in two orographic units, Beskidian Piedmont and Šariš Highlands.

## Discussion

In our study, the survival of heterovegetatively propagated curly birch individuals from different areas of their natural habitat in Slovakia was at the rate of 73% after 28–33 years of growth. The higher mortality occurred in individuals originating from shrub growth. Other phenotypic traits have no significant influence.

Very interesting findings of the initial success of vegetative propagation and the mortality of grafted individuals during orchard establishment were published by Pagan and Paganová^4^. The initial survival depends on the clone genetic makeup, locality of its origin and the calendar year of engraftment^4^. The mean survival of heterovegetatively propagated young curly birch individuals during the three years of repeated grafting (1979, 1980, and 1981) was 63.7%^4^. In some years, ten per cent of clones (the sites of origin) did not survive at all and repeatedly showed 100% mortality.

On the contrary, some grafts from other localities in Slovakia experienced survival of 100% in the first year of their life and the following years. Václav^8^ reported a similar survival rate of 78% of young grafted individuals from Czech localities. Thus, the short and medium-term survival rates can be assessed as relatively favourable. Besides, if the proportion of shrub forms of the parent clones will be reduced when the orchard is established; the progeny survival can be improved further.

Curly birch population in seed orchard retain the most tree-like growth form (96%), with most of the individuals showing a white bark colour (92%). Based on our results, an external manifestation of curly-grained wood seems to be very stable within the heterovegetative progeny. The proportion of individuals expressing curly-grained pattern (though with varying level of quality) within the progeny was 96.4%. Similarly, to curly-grained wood, the bark colour showed high phenotypic stability. According to our results, 99% of progenies originating from parent clones retained the white bark colour of parent clones. Among the originally eight per cent of dark-coloured parent clones, 60% of progenies preserved unchanged dark bark colour. Jawiszczak et al.^24^ studied molecular markers and leaf morphology of two unstable taxa of dark barked *Betula obscura* Kotula ex Fiek and *B. pendula* var. *carelica* with figured wood. Authors revealed a close relationship between the two rarely occurring birches, in the sense that both taxa represent an intraspecific variation of *B. pendula.* They are not intraspecific variations of *B. pubescens*, nor hybrid species of *B. pendula* and *B. pubescens*. The intraspecific variability of both taxa can explain the observation in our study that up to 60% of progenies preserved unchanged bark colour from the original eight per cent of naturally occurring dark-coloured parent clones. It can be hypothesized that the white colour of the bark appears to be the dominant feature, while the dark colour of the bark disappears as the growth pattern and climatic conditions change.

Obtained results are very valuable. Already in 1958, Sarvas has described the vegetative progeny of the triploid curly birch and stated that the curly-grained wood has not been preserved in all the individuals^26^. The basic cause of the curly-grained wood formation and the pattern of curliness inheritance have been hypothesized. The inheritance of the grained wood structure depends on the species and sometimes on the provenance of the species^27^. Whether the inheritance patterns are due only to genetics or the environment too is difficult to prove. Research on the genetic patterns of grained wood formation and reproduction of individuals with figured wood often failed.

For instance, vegetative propagation of figured form of *Acer rubrum, Liriodendron tulipifera* and *Juglans nigra* did not produce trees with figured wood (Bailey 1948)^28^. Propagation of curly-grained and bird's eye sugar maple (*Acer saccharum*) by cuttings have been unsuccessful^29^. Micropropagation of wavy grain *Acer pseudoplatanus* brought not convincing results^30^. Reproduction by organ cultures was not very successful (high initial contamination of buds and mortality) either. In the subsequent population of in vitro regenerants, only one to seven per cent retained the wavy-grained wood structure. Ewald and Naujoks^31^ tested wavy-grained wood structure in the vegetatively propagated trees of *A. pseudoplatanus*. The wavy-grained structure began to appear at around ten years of age, the question is whether it occurs in the entire population.

Bäucker and Liesebach^32^ propagated wavy grain *A. pseudoplatanus *in vitro from grafted individuals. The authors developed a reliable micro-propagation procedure based on nuclear microsatellite markers that enable routine selection of clones for cultivation (clones with retention of the figured pattern). A similar micro-propagation procedure was developed for *Acer macrophyllum*, which identifies potential markers of early chemical differences between clones with figured and non-figured wood structure^33^. The reason for early chemical differences (cell walls with the content of two different components, m/z_69 and m/z_298) remains unclear. The author states that in vitro procedure does not ensure the formation of similar figured wood structures and that propagated individuals in the age of 10–15 years often do not manifest any signs of figured wood formation.

The preservation of curly-grained wood patterns and bark colour of heterovegetatively propagated curly birch in our orchard was very high. The potential of curly birch to retain key properties in Slovak natural conditions seems to be high and applied approaches of heterovegetatively propagation seems to be correct. This indicates the high conservation and/or commercial potential of orchards.

Although a heterovegetative propagation method was undertaken, which ensured the constant nutrition of individuals with a strong root system (note that in the case of autovegetative propagation from adventitious roots, an adequately large, well-branched root system never develops), a large proportion of progenies manifested shrub growth form. The individuals created several stems of lower diameter branching above the surface. In total, twelve per cent of progenies retained the ability to maintain a strong monopodial growth stature manifested by a single continual stem (i.e. 88% of tree parent forms were converted to progeny shrubs). The highest proportions of retained tree forms were recorded in four orographic units and ranged from 21 up to 30% (Busov, Torysa Highlands, Skorušinské Hills, Šariš Highlands).

This finding can be partly explained by the fact that collection of scions was not always done from the apical, dominant part of the tree crown (Lukáčik, pers. comm.), but from lower lateral branches easily reachable from the ground. The vegetative material taken from these parts of the crowns shows so-called "plagiotropic growth". It continues to grow without significant apical dominance and more frequently creates multiple branches and at the same time has a significantly lower growth rate. The growth form and rate of grafted plants are not comparable to generative seedlings and may therefore not be ideal for veneer production^31^.

The low apical dominance and high cytokinin content especially in the *B. pendula* var. *carelica* (5.2–8.9 times higher than that of *B. pendula*) would provide another possible explanation for the significant shrubby growth of vegetatively propagated progeny^34^. Both factors may have a synergistic effect, as evidenced by our finding that up to 96% of progeny retained initial shrub growth form of parent clones and only four per cent attempted to produce a tree growth form. Such proportion is very small in comparison with 35% proportion of progeny with tree growth form transformed from parent clones of curly birch with shrub growth reported by Saarnio^35^. Shrub growth forms of curly birch are especially typical of the southern limit of its natural occurrence in Karelia^6^. Overall, the ability to retain or produce tree growth form among heterovegetatively propagated progenies in the orchard was low, which had a negative impact on the commercial value of progenies and is undesirable for gene conservation.

General stability of forms of curly-grained wood manifested on trunks as bulges is small. The correspondence and coincidence analyses proved significant instability (discrepancy rates prevails the coincidence ones in a ratio of 2:1). Our results point out that the most stable forms are shallow and dense types of bulges (43 and 37% rates of coincidence). On the other hand, sphere type of progeny bulges showed the lowest correspondence to parent clones and it was almost absent in the next vegetatively propagated generation.

The high variance of grained wood types was reported, for example, by Jermakov^36^. He used a transplant of the curly birch bark on downy and on European white birch. In the second year after intergrowth, places under the transplanted bark began to show grained wood structure in a large variability range, intensity, form of the decorative texture of individuals within the same hybrid group.

High variability and instability of bulge forms cannot be explained by the utilization of heterovegative propagation method. A case in which a rootstock and a scion would interactively affect the formation of wood and bulges has never occurred; each part of the grafted individual grows according to the patterns of its identical part^1,13^. This is documented by the fact that if only part of the bark or the whole ring of the curly individual was used, the bulges and the grained wood was formed exclusively at the place of transplantation. The curliness has spread radially, never up- or downwards, while at the same time the effect of the transplanted tissue (ring) has been amplified, not weakened.

The physiological mechanism of curly grained wood formation is complicated and it is strongly affected by a large number of environmental factors that manifest an extraordinary variability in the very heterogeneous natural environment in Slovakia. Genetically strongly controlled polymorphism predisposes curly birch to morphological and environmental plasticity^3^. Its transient types of habitus (tree-like or shrub growth forms, light or dark bark colour) and the high holding capacity of secondary metabolites provides indirect evidence that the curly birch populations are very young on the Eurasian continent. At present, curly birch populations are still significantly transforming. The base population of curly birch in Slovakia growing under the very diverse environmental niches, along with the complex conditions and mechanisms behind the formation of grained wood structure and strong polymorphism are probably the reasons why there was no genetically significant link to one type (form) of grained wood, which would transfer dominantly to vegetatively propagated individuals. Individuals with spherical types of bulges have become the most vulnerable to conservation in this respect.

Curly-grained birch wood is highly valued^37^. The base population of curly birch in Slovakia fulfils the quality and dimension standards required for veneer production^4^. Our results show that the highest proportions of high stem quality parent clones (trees forms with valuable shallow and dense type of bulges) originated from the Low Beskids region. High stem quality trees were absent in half of the surveyed orographic units. Interestingly, in all except one of these units, one to three individuals within the progeny showed a high quality of stems and in two orographic units, high quality of progeny stems attained even nine and 20% (Beskidian Piedmont and Šariš Highlands).

In the majority of studied orographic units with the presence of high-quality mother trees, the proportion of high-quality progenies, however, significantly decreases after the transfer to the orchard. Only about twelve per cent of progenies originating from clones of tree growth form remained trees. At the same time, the growth vitality and rate of grafted individuals are not comparable to generative seedlings' growth^8,17,31^. Heterovegetative propagation seems to be inefficient to produce sufficient sizes of the stems for decorative veneer. Besides, the correspondence analyses proved that the forms of bulges are significantly unstable. Consequently, the stem technical quality (defined as combinations of growth forms and type of bulges) is comparably unstable. Especially, the strong negative association in small-item combinations between the parent trees and progenies was registered and documented by a very large discrepancy rate (95%).

On the other hand, a situation in the veneer category is more favourable. The heterovegetative propagation (grafting) showed to be relatively efficient in retaining the dense and shallow type of bulges. At the same time, these types of bulges are the most frequent. The proportion of progenies with the dense and shallow type of bulges represented 70% and 84% respectively. Correspondence analyses in the veneer category (combinations) showed a negative association with a discrepancy rate of 59%, indicating only a medium strength of association, more favourable than the small item category.

The value of curly birch timber can vary considerably^38^. There could exist an increasing demand for grained branch wood, depending on the quality, length, and diameter of the stem/branch part. The popularity of this wood has increased recently, curly birch has been more often used for small handicraft items and business gifts^39^. According to our findings, production of various small wooden items should be guaranteed at least. The larger coincidence of veneer quality stems provides some positive hints about the commercial value and possible industrial utilization of orchards. However, careful planning and assessment of the profitability of commercial plantations would be needed.

Investigating the influence of the parental environment on new vegetatively propagated progenies, we found that survival rates and proportions of tree growth forms are almost unaffected by the location and altitude of parent sites and their distance and direction of transfer from an orchard. Only a few, but insignificant tendencies are visible. For example, the proportion and the survival rate of the progenies with shrub growth form increased with distance from the parent's site of origin. Besides, the survival rate increases in the direction of transmission from the north-east to the south-west. Oppositely, the individuals had the lowest survival rate, when transferred from north-west to south-east (Fatra-Tatra region, Inner Western Carpathians).

Interestingly, the longest transfer distance reports Ablajev^40^, who planted generative progeny of curly birch of Belarusian origin in conditions of the continental climate of Uzbekistan. The characteristic patterns of wood and morphological characteristics were fully manifested there. Lyubavskaya^2^, Jevdokimov^41^, and Vetchinnikova et al.^13^ also describe the respective transfer of curly birch populations within Karelia, Belarus, and Russia (in the EW direction, slightly S). They observed a shortened vegetation period and increased fructification after the establishment of experimental plots and seed orchards. The conservation of sufficient genetic value (proportion of curliness, single straight stems) was reported while transferring generative progeny of curly birch populations to northern relatively long distances in Finland^42^. The majority of heterovegetatively propagated individuals transferred from the locality Jasénka (Beskydy, Czech Republic) to locality in Kostelec (Czech Republic; 500 km transfer in north-west direction) developed grained-wood structure (61% of the individuals^17^). All studies documented, similarly to our results, that substantially larger distances and/or different transfer direction in boreal and temperate climate zone have insignificant negative effects on survival, curliness occurrence or growth form proportions.

The weak relation of parent environment and progeny properties may partly result from the methodical limits of our study that have the nature of the case study. Many scientific works report that, except for the genetic factors, the formation of grained-wood is affected also by the environmental conditions and vitality status of the tree. To repeat clonal propagations in other localities with different environmental settings in such wide design as in our study is very costly. Thus, unique conditions of seed orchard ʻŠírinyʼ allow only partial understanding of phenotypic variation in wood formation and growth processes of curly birch. However, the research facility still provides valuable findings on the effectivity of heterovegetative propagation regarding the success of conservation effort or the establishment of commercial plantations under the similar natural conditions as are characteristic of a particular orchard. Still the orchard allows rapid obtaining of breeding material for possible cultivation for practical purposes.

## Conclusion

Curly birch in Slovakia remains rarely naturally occurring tree, which occurrence has been endangered because of human-induced changes in land use, climate change and discontinuous populations. The protection of endangered curly birch populations through heterevegetative propagation was evaluated as a very promising conservation method. The registered medium-term survival of grafted individuals was acceptable and retention of curly-grained wood formation was high. The curly-grained wood structure is heritable and thus clonally efficiently achievable (only 3.5% of grafted individuals showed no occurrence of figured wood structure).

Still, the phenotypic expression of curliness manifested on the trunks as bulges showed a lower degree of stability (coincidence) between the parent clones and heterovegatively propagated progenies. We documented high variation in growth forms and stem technical quality among clones and progenies as well. In case of propagating the curly birch artificially, the incidence of individuals with transformed stature toward shrub growth form and presence of shallow and dense forms of grained wood with higher frequency should be considered. The high instability of growth forms and survival of progenies can likely be improved by a more careful selection of scions from apical crown parts of parent clones. The varying degree of phenotypic transmission, manifested in progeny as changes of stature, and shape of bulges in comparison to parent clones, possibly relates to high morphological and environmental plasticity of the phylogenetically young populations of curly birch in Europe.

The survival and growth forms of heterovegetatively propagated curly birch were insignificantly affected by environmental conditions on parent clone sites. That means parent clones from different spatial locations on considered case study area are equally ecologically or economically valuable. If the future orchards would be established in similar environmental conditions in Slovakia, the selection of clones according to locations, direction and altitudes of parent sites probably will not significantly improve the gene conservation or economical value of progenies.

The popularity of curly-grained wood has increased recently, curly birch wood has been traditionally used for decorative veneer and precious furniture production, but it has been also increasingly used for the production of small handicraft items and business gifts. In this regard, the technical quality of progeny stems was manifested as unstable within the vegetative propagation (because of high growth and bulge forms instability). Still, the production of various small wooden items should be guaranteed. The larger coincidence of veneer quality stems among clones and progenies provide some positive hints about the possible effectivity of the commercial utilization of orchards. However, careful planning and assessment of the profitability of commercial plantations are needed. Overall, the establishment of heterovegetative orchards can be recommended as a very promising method for the long-term protection of endangered populations of curly birches, which, in addition to their great biological and ecological value, also have considerable commercial potential.

## Methods

### Study site (locality of seed orchard)

Seed orchard ʻŠírinyʼ is in the central part of Slovakia, in Zvolen Basin, cadaster of Budča village in altitude of 430–450 m asl (Fig. 1). Southeast exposed area of seed orchard expanses 3.38 ha. One-third of the area is flat and two-thirds have slope inclination in the range from 3 to 11°. Locality ʻŠírinyʼ belongs to the area of warm climate, particularly to warm slightly moist zone (district) with cold winter^23^. The mean monthly temperature ranges from 16 to 18 °C in July and is below − 3 °C in January. Mean annual precipitation sum ranges from 550 to 600 mm. Location in basin necessitated higher annual frequency of fogs in the range from 80 to 100 days. Bedrock consists mainly of pyroxenic and ambifolic-pyroxenic andesites. Eutric Cambisols associated with Stagni-Eutric Cambisols prevail. The humus layer represents 1.8–2.3%. The moderately moist soils are characterized by high retention capacity, medium permeability, and neutral to slightly alkaline reaction [pH (H_2_O) of 7.3–7.8].

### Establishment of seed orchard

The fenced curly birch seed orchard was established in autumn 1982. Mechanized site preparation by the deep ploughing and defending was done before planting the orchard. To establish the seed orchard, standard procedures of grafting were followed. All rootstocks were of the same local origin (locality ‘Záhorie’), the species: *B. pendula*, and in the year of grafting (1979) they were three years old. Curly birch (*B. pendula* var. *carelica*) is most often considered to be a variety or genetic variant of silver birch (*B. pendula*)^24^. The scions (cuttings) of *B. pendula* var. *carelica*, were collected in localities with natural occurrence of curly birch in Slovakia (Fig. 1) and grafted in early spring (February, March) during the years 1979 and 1980. Grafts were planted in the seed orchard two–three years since grafting (according to the growth performance of particular scions). Later, the plantings were sown with grass seeds and regularly mowed. Each clone, from which scions were collected, was described in the sense of its qualitative and quantitative characteristics, photo documentation, and basic facts about the locality. Seed orchard is composed of 84 clones planted in ten blocks with the random layout of a position of individuals. Each clone (origin) is represented in the block once. Altogether, 840 individuals (10 individuals per clone) are planted at spacing 5 × 5 m. Thirty-one grafts died due to lack of precipitation in 1983. In spring 1984, plants grafted to complete absent individuals in 10 established blocks and 60 newly grafted plants to establish 11th spare block were planted. Orchard continued to be gradually completed by new grafts and reached the final number of 102 clones and the total number of 939 individuals in 1988. Since then the composition of the orchard did not change.

### Empirical material

The base population of curly birch in Slovakia expresses high variability of growth forms and manifests externally various signs of patterned wood (bulges on the stem). Among the growth forms (long-stem trees, short-stem trees, or shrubs), the most frequent is the short-stem tree and shrub growth form. In the growth form of single long-stem curly birch trees, trees with the spherical shape of bulges more frequently occur than trees with the external shallow or dense shape of bulges (Fig. 2). Additional forms, not reported from other areas of curly birch natural occurrence, can be found in Slovakia, such as a form with the dark colour of the bark or bird-eye form manifesting clusters of dormant buds^4^. Based on the national survey conducted in the 80 s, the long-stem trees generally reach the height of 10–11 m and the corresponding diameter 16–22 cm. The short-stem trees reach the mean height of 6–7 m and the mean diameter of 14–15 cm. The average growth performance of shrubs corresponds to the height of 5–6 m and the diameter of 12–13 cm.

### Evaluation of progenies in seed orchard ʻŠírinyʼ

The dataset used in this study consists of 95 clones (origins) representing the occurrence of the base population of curly birch in Slovakia (Table 1). In the established seed orchard, 896 vegetatively propagated progenies originating from predominantly parent individuals of tree growth form were examined. We recorded the qualitative characteristics of curly birch progenies. Individuals overgrowing the rootstock were excluded from the evaluation. The following characteristics were recorded:

**Survival**—living/dead individual, according to the presence or absence of individuals in the 2015 census.

**Growth form**—tree/shrub. Tree growth form includes single long- and short-stem individuals. Shrub growth form includes multiple stems or single short-stem individuals with several stems, which diameter size reaches from 1/3 up to 1/2 of the single stem diameter.

**Bark colour**—white/dark.

In addition to survival, growth form, and bark colour, we assigned every individual to one of four types of bulges visible on the stem surface. We consider the external manifestation of bulges as an indirect indicator of patterned wood structure (Fig. 2).

**Type of bulges**

- Ring type—with thick and tight rings, bulges are approximately twice as wide as the diameter of the evaluated stem or branch.
- Dense type—dense agglomerated protuberances or nubs manifested on more than one-third of the stems or branches.
- Shallow type—fine wavy forms on the stem or branch.
- Sphere type—spherical (globular) shape of bulges; unlike in ring type, the diameter of bulges is larger than their height.

For categorization of individuals into various types of bulges in case of tree growth form, a single stem was evaluated. In the case of shrub growth form, the three thickest branches were evaluated.

**Technical quality of stems**

The eight combinations of growth forms and type of bulges (two growth forms x four bulges types) were separated into two groups: (i) combinations allowing veneer production and (ii) combinations utilizable only for the production of small decorative items. Both groups (denoted as a veneer or small items) were utilized in the subsequent analysis to assess the relationships between stem technical quality and progeny survival or in two separate correspondence analyses between parent and progeny individuals in each group testing the stem technical stability in time.

### Data analysis

To evaluate the differences in the survival rate of progenies in seed orchard according to categorical characteristics of parent clones (growth form, bark colour, the shape of bulges and stem technical quality categories), we processed data statistically by two-way contingency tables. We used the Pearson Chi-square test to confirm the relationships at the significance level of 0.05. If the tests indicate significant differences between the observed and expected frequencies used for calculation of Chi-square statistics (expected frequencies are theoretical frequencies in individual cells assuming that there is no relationship), the statistical evidence about the significant influence of analysed trait of parent clone on the survival rate of progenies was provided.

Similarly, to statistically explore the stability of phenotypic traits and stem technical quality among parents and progenies (growth form, bark colour and shape of bulges and the technical quality combinations), we used correspondence analysis. Based on the overall Chi-square statistic, the existence of correspondence between progenies and parent clone traits were tested at the significance level of 0.05. If the tests did not prove a significant association between the parent clones and progenies, the empirical evidence about medium instability of the analysed trait was obtained. Moreover, nature (positive or negative) and strength of associations was studied through the calculation of coincidence and discrepancy rates based on the on and off-diagonal frequencies in classification tables. The discrepancy rates were tested by standard Student's test to confirm the significance of deviations from zero. The traits showing significant association with high coincidence and low discrepancy are interpreted as very stable, and oppositely the traits with the confirmed significant association, low coincidence and high discrepancy rates are interpreted as highly unstable.

To quantify the expected environmental influence on progenies, four environmental variables were selected to assess the effect of environmental conditions of parent clone localities on the survival rate and proportions of progeny growth form. The information about spatial patterns of most successful parent clones on the considered study area is expected to be important for the effective establishment of future gene pools or commercial plantations. The effects of distance between the locality of the parent tree and seed orchard and the effect of parent locality altitude were evaluated by generalized linear models. Survival rates and proportions of progeny shrubs and trees as dependent variables entered to the analysis after transformation by logit-link function.

The effects of the geographical origin of parent clones represented by geomorphological sub-province of Carpathians (Inner Western, Outer Western, and Outer Eastern) and the transfer direction from parent locality toward the clonal seed orchard (SW, SSE, SWW, W, No, S, SE) was tested by one-way ANOVA at significance level equal to 0.05. Two separate analysis of categorical independent variables instead of building a complex general linear model was needed due to sample size limitations. Similarly, to regression analyses, the survival and growth form rates were logit transformed to approach the normality assumption. The above analyses were performed using STATISTICA software^43^.

## Data Availability

The datasets generated during and/or analysed during the current study are available from the corresponding author on reasonable request.
